# Changing the Receptor Specificity of Anthrax Toxin

**DOI:** 10.1128/mBio.00088-12

**Published:** 2012-05-01

**Authors:** Adva Mechaly, Andrew J. McCluskey, R. John Collier

**Affiliations:** Department of Microbiology and Immunobiology, Harvard Medical School, Boston, Massachusetts, USA

## Abstract

The actions of many bacterial toxins depend on their ability to bind to one or more cell-surface receptors. Anthrax toxin acts by a sequence of events that begins when the protective-antigen (PA) moiety of the toxin binds to either one of two cell-surface proteins, ANTXR1 and ANTXR2, and is proteolytically activated. The activated PA self-associates to form oligomeric pore precursors, which, in turn, bind the enzymatic moieties of the toxin and transport them to the cytosol. We introduced a double mutation into domain 4 of PA to ablate its native receptor-binding function and fused epidermal growth factor (EGF) to the C terminus of the mutated protein. The resulting fusion protein transported enzymatic effector proteins into a cell line that expressed the EGF receptor (A431 cells), but not into a line lacking this receptor (CHO-K1 cells). Addition of excess free EGF blocked transport of effector proteins into A431 cells via the fusion protein, but not via native PA. We also showed that fusing the diphtheria toxin receptor-binding domain to the C terminus of the mutated PA channeled effector-protein transport through the diphtheria toxin receptor. PA fusion proteins with altered receptor specificity may be useful in biological research and could have practical applications, including ablation or perturbation of selected populations of cells *in vivo*.

## IMPORTANCE

Bacterial toxins that act within mammalian cells have receptor-dependent mechanisms to transport their enzymatic components to the cytoplasmic compartment. By inactivating or otherwise modifying their respective intracellular targets, these intracellular effectors disrupt metabolic pathways and in some cases cause death of the cell. Our results show that the receptor specificity of the transport protein of anthrax toxin may be readily changed, raising the possibility that receptor-redirected forms of protective antigen (PA) and PA homologs may be useful for research and medical applications requiring modification or ablation of designated populations of cells.

## Introduction

Targeting of toxic proteins to specific classes of mammalian cells has been studied extensively in recent years, often with the goal of developing new treatments for malignancies (1). One approach to targeting involves replacing the receptor-binding domain of a toxin with a heterologous protein, such as a growth factor or antibody that binds to a specific cell-surface receptor. Another approach is to link a heterologous protein to an altered form of the toxin in which the native receptor-binding function has been disrupted. We used the latter approach to redirect the receptor specificity of the transport moiety of anthrax toxin (ATx) to either one of two heterologous receptors.

Anthrax toxin is an ensemble of three large proteins: protective antigen (PA) (83 kDa), lethal factor (LF) (90 kDa), and edema factor (EF) (89 kDa) (2). LF and EF are enzymes (“effector proteins”) that modify substrates residing within the cytosolic compartment of mammalian cells. LF is a metalloprotease that cleaves most members of the mitogen-activated protein (MAP) kinase kinase family, and EF is a calmodulin- and Ca^2+^-dependent adenylyl cyclase, which elevates the level of cyclic AMP (cAMP) within the cell (3–5). PA is a receptor-binding transporter, which is capable of forming pores in the endosomal membrane (2, 6). These pores mediate the translocation of EF, LF, or various fusion proteins containing the N-terminal PA-binding domain of EF or LF across the endosomal membrane to the cytosol (7).

ATx action at the cellular level is initiated when PA binds to either one of two receptors, ANTXR1 and ANTXR2 (8, 9), and is activated by a furin class protease (10). The cleavage yields a 20-kDa fragment, PA_20_, which is released into the surrounding medium, and a 63-kDa fragment, PA_63_, which remains bound to the receptor. Receptor-bound PA_63_ spontaneously self-associates to form ring-shaped heptameric (11) and octameric (12) oligomers (prepores), which are capable of binding LF and/or EF with nanomolar affinity (13, 14). The resulting heterooligomeric complexes are endocytosed and delivered to the endosomal compartment, where acidic pH induces the prepores to undergo a conformational rearrangement that enables them to form pores in the endosomal membrane (2). These pores serve as protein translocases, which unfold bound LF and EF molecules and transport them across the endosomal membrane, where they refold and modify their respective intracellular targets.

Both PA receptors—ANTXR1 (also called TEM8) and ANTXR2 (also called CMG2)—are type 1 membrane proteins containing a von Willebrand/integrin A (VWA) MIDAS domain (2). Within PA, both domain 4, the so-called receptor-binding domain, and domain 2, the pore-forming domain, participate in binding to the MIDAS domains of the receptors (15). ANTXR1 and ANTXR2 have different affinities for PA (16, 17), but both of these receptors bind PA in a manner that allows it to be proteolytically activated and to oligomerize and both receptors mediate trafficking of prepore-effector complexes to the endosomal compartment and translocation across the endosomal membrane.

**FIG 1:**
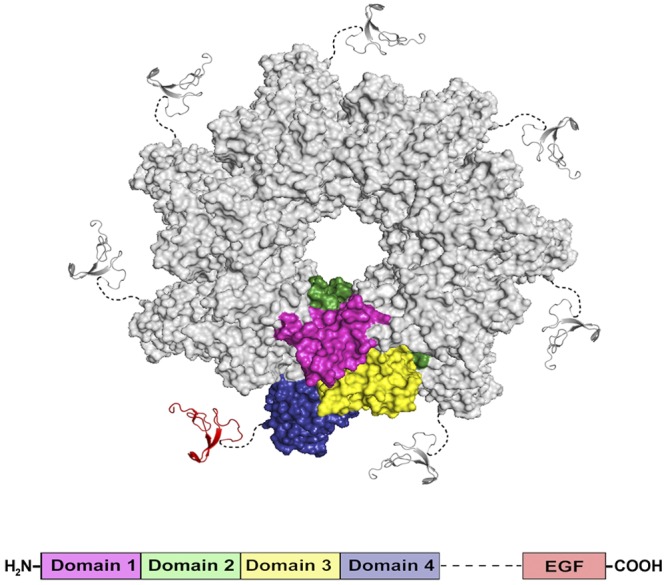
Composite representation of the heptameric prepore formed by PA_63_ (Protein Data Bank [PDB] accession no. 1TZO) with EGF (PDB accession no. 1JL9) linked to the C terminus. An axial view of the heptameric prepore is shown, with domains 1, 2, 3, and 4 in a single subunit of PA_63_ colored magenta, green, gold, and purple, respectively. EGF is in red. Broken lines represent an 8-amino-acid linker (SPGHKTQP) connecting the N terminus of EGF to the C terminus of PA_63_.

In this study, we ablated the receptor-binding activity of PA by mutating two residues within domain 4 and then fused each of two heterologous receptor-binding proteins, human epidermal growth factor (EGF) or the receptor-binding domain of diphtheria toxin (DTR), to the C terminus of the mutated protein (Fig. 1). Both of the resulting fusion proteins mediated the entry of effector enzymes, and entry was dependent in each case on the cellular receptor recognized by the receptor-binding polypeptide appended to PA.

## RESULTS

**FIG 2:**
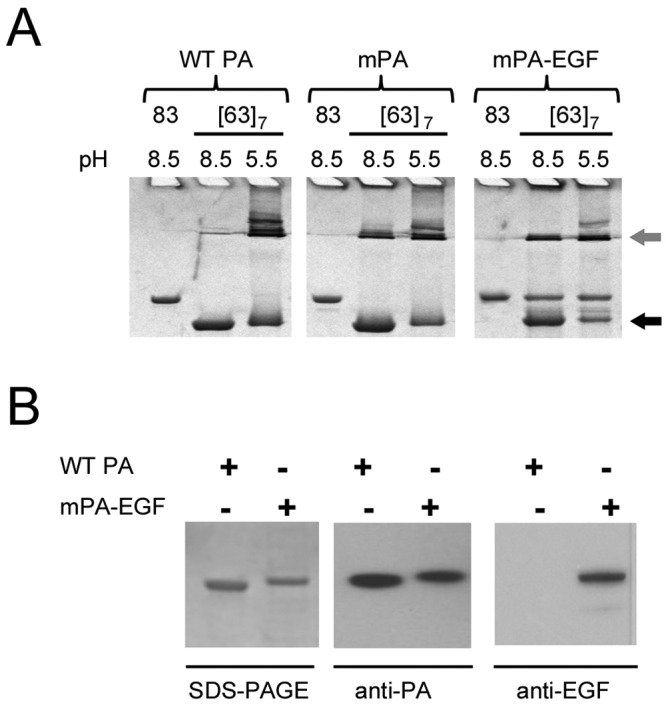
Characterization of purified mPA-EGF. (A) Conversion of PA_63_ oligomers from the SDS-dissociable prepore state (black arrow) to the SDS-resistant pore state (gray arrow) at different pH values. Samples (5 µg) of native (83 kDa) and proteolytically activated ([63]_7_) forms of WT PA, mPA, and mPA-EGF were separated by SDS-PAGE and visualized by Coomassie blue staining. (B) Western blot analysis with anti-PA and anti-EGF antibodies demonstrating the presence of both the PA and EGF epitopes in the purified fusion protein.

We introduced two mutations, N682A and D683A, into PA to ablate its native receptor-binding function (18), and expressed the mutated protein (mPA) in *Escherichia coli* BL21(DE3). The purified product failed to promote entry of LF_N_-DTA into either CHO-K1 cells or A431 cells at the highest concentration tested (10 nM), as measured by the inhibition of protein synthesis in the presence of LF_N_-DTA. LF_N_-DTA is a fusion between LF_N_, the N-terminal PA_63_-binding domain of LF, and DTA, the catalytic domain of diphtheria toxin. The DTA moiety catalyzes the ADP-ribosylation of eukaryotic elongation factor 2 (eEF-2) within the cytosol, blocking protein synthesis and causing cell death (19, 20). The proteolytically activated form of mPA, mPA_63_, was able to form SDS-resistant, high-molecular-weight aggregates, characteristic of pores, although the pH dependence of pore formation was somewhat altered (Fig. 2A).

Having demonstrated that the N682A/D683A double mutation blocked the receptor-binding function of PA, we fused human EGF to the C terminus of the mutated protein (mPA-EGF). Purified monomeric mPA-EGF was stable and ran slightly slower than native PA on SDS-polyacrylamide gels, consistent with its higher molecular weight (Fig. 2B). Western blots showed that the product reacted with both anti-PA and anti-EGF antibodies. Also, it was shown that the mPA_63_-EGF fragment derived by trypsin treatment formed high-molecular-weight aggregates on SDS-polyacrylamide gels similar to those seen with mPA_63_ (Fig. 2A).

**FIG 3:**
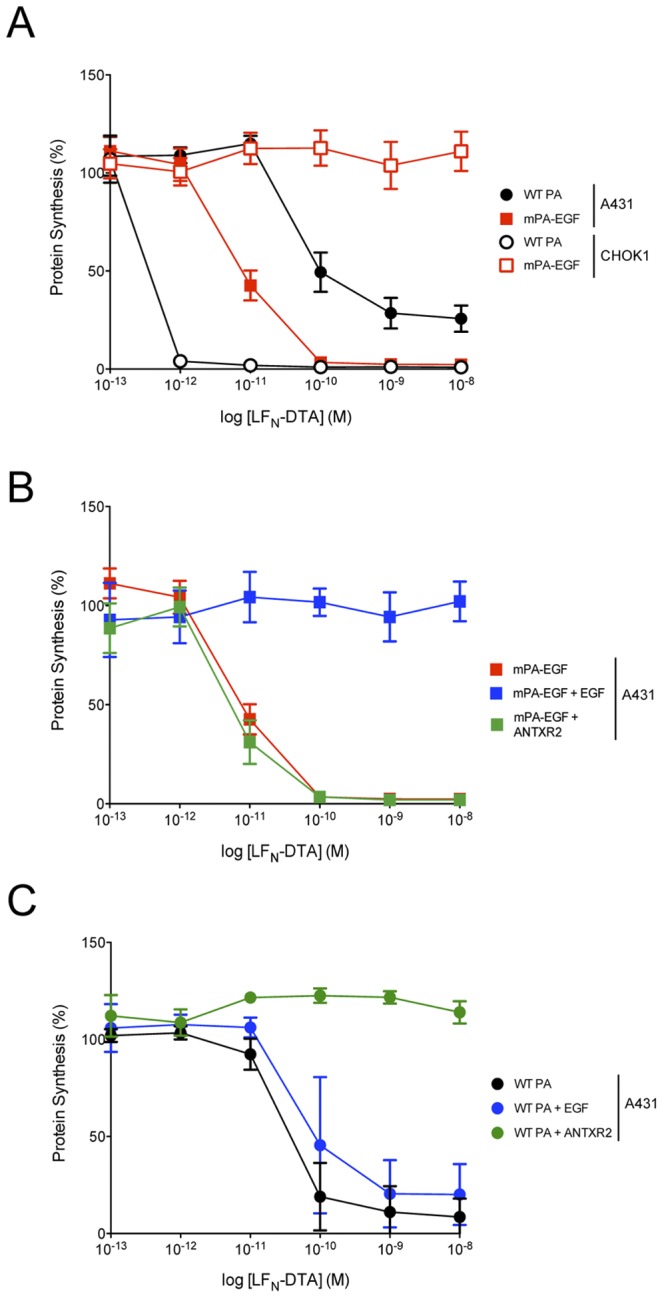
Cytotoxicity assays demonstrate receptor-specific cell targeting of mPA-EGF. (A) A431 or CHO-K1 cells (3.5 × 10^4^) were incubated with 10 nM PA or PA variant plus LF_N_-DTA at the concentrations indicated. After a 4-h incubation (A431 cells) or overnight incubation (CHO-K1 cells), the medium was replaced with medium containing 1 µCi of [^3^H]leucine/ml. Following a 1-h incubation, incorporated [^3^H]leucine was determined by scintillation counting. (B and C) Assays were performed as described above for panel A, but soluble EGF (500 nM) or the PA-binding VWA domain of ANTRX2 (ANTRX2; 100 nM) was present during a 4-h incubation with A431 cells. Each point on the curves represents the average of three experiments.

A431 cells, which express high levels of the EGF receptor (EGFR) (21, 22), were killed by LF_N_-DTA (50% effective concentration [EC_50_] of ~10 pM) in the presence of mPA-EGF, whereas CHO-K1 cells, which do not express the EGF receptor, were not killed (Fig. 3A). Wild-type PA also mediated the inhibition of protein synthesis in A431 cells, but a high concentration of LF_N_-DTA (EC_50_ of ~100 pM) was needed, suggesting that these cells express a lower level of ANTXR1, ANTXR2, or both. A translocation-deficient PA mutant, PA^F427H^ (23), did not mediate killing of either A431 or CHO-K1 cells (data not shown).

If the entry of LF_N_-DTA into A431 cells mediated by mPA-EGF were dependent on binding to the EGF receptor, then addition of free EGF should compete for binding and block toxicity. As shown in Fig. 3B, a 50-fold excess of EGF completely protected the cells from the cytotoxic effects of LF_N_-DTA, whereas the same concentration of the PA-binding VWA domain of ANTXR2 had no effect. In contrast, cytotoxicity mediated by wild-type PA on A431 cells was ablated by the ANTXR2 domain, but it was not inhibited to a significant degree by EGF (Fig. 3C).

**FIG 4:**
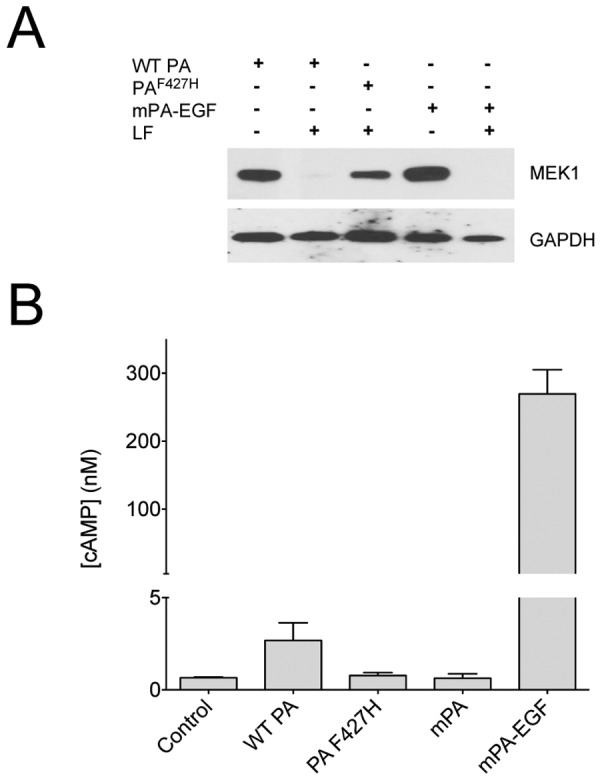
mPA-EGF transports LF and EF into EGFR-bearing cells. (A) A431 cells (1 × 10^6^) were treated with 100 nM LF plus 10 nM PA or PA variant for 3 h. Cell lysates were prepared, fractionated by SDS-PAGE, and transferred to a PVDF membrane, and MEK1 cleavage was evaluated by Western blotting with anti-MEK1 antibody. For a control, GAPDH was monitored with anti-GAPDH antibodies. (B) A431 cells (3.5 × 10^4^) were exposed to 50 nM EF plus 10 nM PA or PA variant for 1 h. A competition enzyme-linked immunoassay was performed to detect the intracellular concentration of cAMP, based on a standard curve, following the protocol of the manufacturer (Cell Signaling Technology). The column labeled “Control” corresponds to A431 cells treated with EF in the absence of PA. Each bar represents the average of experiments performed in quadruplicate.

We tested the ability of mPA-EGF to translocate LF and EF, the native effector moieties of anthrax toxin, into A431 cells. LF inactivates mitogen-activated protein kinase kinases (MEKs) by cleaving near their N termini (3, 5), and we measured LF entry by Western blotting of cell lysates with an anti-MEK1 antibody after incubating cells with LF plus PA or a PA variant. MEK1 was cleaved completely with LF in combination with PA or mPA-EGF, but not in combination with the translocation-deficient mutant PA^F427H^ (Fig. 4A). We measured entry of EF using an enzyme-linked competition assay to determine the intracellular level of cyclic AMP (cAMP) and observed a 400-fold elevation of cAMP when mPA-EGF was used as the transporter (Fig. 4B). This level was ~100 times higher than that observed when wild-type PA (WT PA) was used as the transporter. The level of cAMP when mPA or PA^F427H^ was used as the transporter was identical to the background level. The strong elevation observed with mPA-EGF was likely due in part to the high level of EGF receptor (EGFR) on the A431 cells. The fact that cleavage of MEK1 was complete when LF was delivered via WT PA or mPA-EGF, whereas the amount of cAMP generated by EF delivered by mPA-EGF was vastly greater than when EF entered via WT PA may reflect differences in reaction kinetics and the levels of substrates of the two effectors within cells.

We prepared a second fusion protein, in which the 150-residue receptor-binding domain of diphtheria toxin (DTR) was fused to the C terminus of mPA. The purified mPA-DTR fusion reacted with both anti-PA and anti-diphtheria toxin antibodies (see Fig. S1A in the supplemental material) and retained the ability to oligomerize and form pores after activation. The activated form bound and translocated LF_N_-DTA in a planar bilayer system (Fig. S1B). The mPA-DTR variant delivered LF_N_-DTA into CHO-K1 cells, inhibiting protein synthesis, and soluble DTR competitively blocked this inhibition (Fig. S1C).

## DISCUSSION

Viewing PA in broad perspective as a vehicle for delivering proteins to the cytosolic compartment of mammalian cells, one is struck by its adaptability. The ability of PA to transport two structurally disparate enzymes, LF and EF, into cells suggested that it might be capable of delivering heterologous proteins, and indeed, delivery of several such proteins and peptides has been demonstrated following their fusion to the PA_63_-binding domain of LF (24–30). A second mode of adaptability is illustrated by studies in which the furin activation site within PA was replaced with sites specific for other proteases for the purpose of tumor targeting (31, 32). The current study demonstrates a third mode of adaptability, namely, that the protein transport activity of PA can be readily channeled through heterologous cell-surface receptors.

mPA-EGF was able to transport LF, EF, and the LF_N_-DTA fusion protein to the cytosol, suggesting that the essential oligomerization and transport functions of PA were not perturbed by channeling entry through surrogate receptors. One of the surrogate binding domains examined, DTR, performs an analogous function in an unrelated toxin (33), whereas the other, EGF, has no relationship to bacterial toxin action, as far as we know. Both of these proteins bind to receptors that, like ANTXR1 and ANTXR2, internalize their ligands and traffic them to an acidic intracellular compartment. It is likely that entry into an acidic compartment is essential for proper functioning of PA fusion proteins, because (i) acidic intravesicular pH plays a crucial role in promoting conversion of the PA prepore to the pore (6, 7) and (ii) the pH gradient across the endosomal membrane is essential for protein translocation (34).

The decision to fuse surrogate receptor ligands to the C terminus of mPA, instead of replacing domain 4 with these ligands, was based on results indicating that domain 4 stabilizes the prepore (35). Domain 4 must pivot away from domain 2 to allow the pore-forming loop to be relocated to the base of the structure, so that the transmembrane β-barrel stem of the pore can be formed (35). Weak contact of domain 4 with domain 2 within PA_63_ inhibits this pivoting and prevents premature conversion of the prepore to the pore. Thus, retaining domain 4 in mutated form allowed us to change receptor specificity while minimizing the likelihood that the process of prepore-to-pore conversion would be perturbed.

The pH threshold at which the prepore transitions to the pore can be affected by various factors, including receptor binding. When the native receptors bind to PA, they form contacts with domain 2 as well as domain 4 and thereby stabilize the prepore, causing the pH threshold to be shifted towards a more acidic value. This effect should be obviated by ablating the receptor-binding activity of domain 4. In fact, we found that the mPA prepore, unlike the native prepore, underwent some conversion to pores at pH 8.5 (Fig. 2A) even in the absence of native receptor. It may be possible to identify alternative mutations in domain 4 that eliminate native receptor binding activity while causing less perturbation of prepore stability. A shift in the dependence of chimeric, receptor-redirected forms of PA on pH may cause the prepore-to-pore conversion to occur at an earlier stage of the endocytic pathway and possibly even allow some conversion to take place at the cell surface. Regardless, the potency of the constructs evaluated in the current study suggests that any shift in the pH threshold did not greatly diminish the overall ability of PA to deliver effector proteins to the cytosol.

Redirecting PA-dependent protein transport through heterologous cellular receptors may have applications both in experimental science and medicine. Leppla and coworkers have explored targeting of PA to tumor cells by changing the proteolytic activation site. Modified forms of PA were used to deliver FP59, a cytotoxic fusion protein similar to LF_N_-DTA, to the cytosol of cells enriched in urokinase or matrix metalloprotease (31, 32). Like these proteases, EGFR is enriched on several tumors (36), and thus, mPA-EGF could potentially serve as an alternative means of targeting. Other ligands whose receptors are enriched on target cells would also be candidates for fusion to mPA.

One can envision use of receptor-targeted PA variants to deliver a wide variety of proteins (nontoxic and toxic proteins) to chosen classes of cells. However, fusion to LF_N_ does not render all proteins transportable by PA. Like DTA, the catalytic domains of Shiga toxin and pseudomonas exotoxin A, and some nontoxic proteins, including beta-lactamase, dihydrofolate reductase (DHFR), and ciliary neurotrophic factor, were found to be transported by PA when fused to LF_N_ (24, 25, 30, 37), but LF_N_ fusions of other proteins, including tetanus toxin light chain, botulinum toxin E light chain, acidic fibroblast growth factor, basic fibroblast growth factor, and HIV Tat protein, were not transported. Introduction of an artificial disulfide into the DTA moiety of LF_N_-DTA blocked translocation, as did liganding of LF_N_-DTA and LF_N_-DHFR by adenine and methotrexate, respectively (30). These findings are consistent with a requirement that proteins unfold in order to be translocated through the PA pore, and the propensity to unfold under acidic conditions may therefore be a major determinant of the ability of a protein to be translocated.

In conclusion, despite limitations, the PA-based transport of proteins offers a wide range of adaptability and warrants further study for a variety of applications.

## MATERIALS AND METHODS

### Reagents and chemicals.

Oligonucleotides were from Integrated DNA Technologies (Coralville, IA). Sigma-Aldrich (St. Louis, MO) supplied all chemicals unless noted otherwise. A synthetic human EGF gene, adjusted for *E. coli* expression, was a generous gift from E. Joop van Zoelen (Department of Cell Biology and Applied Biology, Radboud University Nijmegen, Nijmegen, The Netherlands). Soluble EGF was from ProSpec-Tany Technogene Ltd. (East Brunswick, NJ).

### Generation of PA expression plasmids.

The two PA chimeras used in this work, PA^N682AD683A^-EGF (mPA-EGF) and PA^N682AD683A^-DTR (mPA-DTR), were created by overlap extension PCR using a previously generated PA^N682AD683A^ (mPA) gene coding sequence. In both cases, the first PCR step consisted of two reactions. The first reaction used a forward primer, PAFor (For stands for forward) (GATTTAGTAATTC**GAATTC**AAGTACGG), plus either PARevEGF (Rev stands for reverse) (CATTCAGAGTCGCTGTTTGGTTGCGTTTTATG) or PARevDTR (GTTTTATGCCCCGGAGATCCTATCTCATAGCC) as the reverse primer. The PARevEGF and PARevDTR primers contained the EGF and DTR overlapping regions, respectively. In the second reaction, forward and reverse primers were used to amplify the EGF sequence (EGFFor [CATAAAACGCAACCAAACAGCGACTATGAATG] and EGFRev [GGTGGTG**CTCGAG**TCAACGGAGCTCCCACCATTTC]) and DTR sequence (DTRFor [GGCTATGAGATAGGATCTCCGGGGCATAAAAC] and DTRRev [GTGGTGGTGGTGGTG**CTCGAG**TCAGCTTTTGATTTC]) sequences. The PCR-generated DNA fragments were then subjected to a second PCR step using forward primer PAFor in combination with either the EGFRev or DTRRev primer, for PA-EGF and PA-DTR, to stitch and amplify the two fragments together. In both cases, the full-length PCR products encoded EcoRI and XhoI restriction sites (indicated in the primer sequences in bold) in the forward and reverse primers, respectively. The PCR products were restriction digested and cloned into the pet22b expression vector by standard protocols. Each clone also coded for an 8-residue linker (SPGHKTQP) between PA and either EGF or DTR, which is part of the natural linker between the transmembrane and receptor-binding domains of diphtheria toxin.

### Protein expression and purification.

Recombinant wild-type PA (WT PA), PA^F427H^, mPA, mPA-EGF, and mPA-DTR were overexpressed in the periplasm of the *E. coli* BL21(DE3) strain (Invitrogen, Carlsbad, CA). The resulting bacterial pellets were lysed and purified as described previously (6). Oligomeric prepores of WT PA and the various PA variants were produced by limited trypsin digestion at a final trypsin/PA ratio of 1:1,000 (wt/wt) for 30 min at room temperature (RT). The nicked proteins were subjected to anion-exchange chromatography, resulting in the separation of PA_63_ and PA_20_ fragments. PA_63_ spontaneously oligomerized to form prepore.

Purified mPA-EGF and mPA-DTR fusions were characterized by Western blot analysis. PA_83_ variants along with WT PA were subjected to SDS-PAGE and transferred to a polyvinylidene difluoride (PVDF) membrane (Invitrogen, Carlsbad, CA). The membranes were blocked with Tris-buffered saline (pH 7.4) containing 2% bovine serum albumin (BSA) and hybridized with either mouse anti-PA antibodies (diluted 1:4,000) (catalog no. MAB8082; Millipore, Billerica, MA), rabbit anti-EGF antibodies (1:50,000) (catalog no. Ab9695; Abcam, Cambridge, MA), or rabbit anti-DT antibodies (1:20,000) (catalog no. Ab53828; Abcam). Primary antibodies were detected with either goat anti-rabbit IgG (1:20,000) (catalog no. sc-2004; Santa Cruz Biotechnology, Inc., Santa Cruz, CA) or rabbit anti-mouse IgG conjugated to horseradish peroxidase (HRP) (1:10,000) (catalog no. sc-358914; Santa Cruz) with enhanced chemiluminescence (ECL) reagents (Pierce, Rockford, IL).

LF, EF, DTR, and LF_N_-DTA were expressed in *E. coli* BL21(DE3) (Invitrogen), induced with 1 mM isopropyl-β-d-1-thiogalactopyranoside (IPTG) for 4 h, using the Champion pet-SUMO expression system (Invitrogen). Cell pellets were lysed by sonication in lysis buffer (20 mM Tris-HCl [pH 8.0], 150 mM NaCl, 10 mM imidazole, 10 mg lysozyme, 2 mg DNase I, supplemented with a complete Roche protease inhibitor tablet). Following sonication, the lysates were cleared by centrifugation and loaded onto a 3-ml bed volume of nickel-nitrilotriacetic acid (Ni-NTA) agarose (Qiagen, Valencia, CA). The resin was washed with 15 column volumes of wash buffer (20 mM Tris-HCl [pH 8.0], 150 mM NaCl, 20 mM imidazole) and eluted with the same buffer supplemented with 250 mM imidazole. The resulting purified protein was transferred into 20 mM Tris-HCl (pH 8.0) and 150 mM NaCl, and cleaved with SUMO protease (Invitrogen) overnight at 4°C. Uncleaved His-SUMO fusion and SUMO protease were removed by a second round of Ni-NTA chromatography, in which the flowthrough contained the cleaved product of interest.

### SDS resistance.

Exposure to acidic pH causes the structural transformation from PA prepores to pores, which is marked by the presence of SDS-resistant oligomers. WT PA, mPA, mPA-EGF, and mPA-DTR prepores (5 µg) were incubated in pH 5.5 buffer (100 mM KCl, 1 mM EDTA, and 10 mM [each] sodium oxalate, potassium phosphate, and morpholineethanesulfonic acid [MES] [pH 5.5]) or pH 8.5 buffer (20 mM Tris [pH 8.5] plus 150 nM NaCl) for 30 min at room temperature. Each sample was then exposed to SDS sample buffer and resolved by SDS-PAGE. Protein bands were visualized by Coomassie blue staining.

### Cell culture.

The CHO-K1 cell line was from the American Type Culture Collection (catalog no. CCL-61) (Manassas, VA). Cells were maintained in Ham’s F-12 medium supplemented with 10% fetal bovine serum (FBS), 500 U/ml penicillin G, and 500 U/ml streptomycin sulfate (Life Technologies, Inc., Carlsbad, CA). The A431 cell line, also from the American Type Culture Collection (catalog no. CCL-1555) was grown in Dulbecco’s modified Eagle’s medium, with 10% FBS, 500 U/ml penicillin G, 500 U/ml streptomycin sulfate, and 1 mM sodium pyruvate (American Type Culture Collection).

### Cytotoxicity assays.

Protein synthesis inhibition was used to measure the ability of WT PA and its derivatives to deliver LF_N_-DTA to the cytosol. CHO-K1 and A431 cells (3.5 × 10^4^ per well) were exposed to six 10-fold serial dilutions of LF_N_-DTA (starting with 10 nM) in combination with one of the PA_83_ variants (10 nM). The cells were incubated either for 4 h (A431) or overnight (CHO-K1) at 37°C. Toxin-containing medium was removed, and the cells were incubated for 1 h at 37°C with leucine-deficient medium supplemented with 1 µCi of [^3^H]leucine/ml (PerkinElmer, Billerica, MA). The plates were washed twice with cold phosphate-buffered saline (PBS) and protein synthesis was measured by the amount of [^3^H]leucine protein, as determined by scintillation counting. Percent protein synthesis was plotted versus the log concentration of LF_N_-DTA where each bar represents the average of three experiments.

Competition experiments were performed as described above but with a 50-fold molar excess of soluble EGF (Prospec, East Brunswick, NJ) or 10-fold excess of DTR to compete with mPA-EGF and mPA-DTR, respectively. Control experiments were also performed with a 10-fold excess of the PA-binding VWA domain of ANTRX2 (ANTHRX2), which was produced recombinantly as described previously (16).

### MEK cleavage.

Translocation of LF to the cytosol of A431 cells was monitored by Western blotting against cell lysates for mitogen-activated protein kinase kinase 1 (MEK1). A431 cells (1 × 10^6^ cells) were exposed to lethal toxin (10 nM PA_83_ variant and 100 nM LF) for 3 h at 37°C. The cells were harvested in 100 µl of Tris-buffered saline (20 mM Tris-HCl, 150 mM NaCl [pH 7.4]), suspended in SDS-PAGE sample buffer, and immediately incubated at 100°C for 20 min. The lysates were resolved by SDS-PAGE and transferred to a PVDF membrane (Invitrogen). The membranes were blocked with Tris-buffered saline (pH 7.4) containing 2% BSA and hybridized with either anti-MEK1 antibodies (1:1,000) (catalog no. Ab32071; Abcam) or anti-glyceraldehyde-3-phosphate dehydrogenase (anti-GAPDH) antibodies (1:2,500) (catalog no. Ab9485; Abcam). Primary antibodies were detected with goat anti-rabbit IgG conjugated to HRP (1:20,000) (catalog no. sc-2004; Santa Cruz) and ECL reagents (Pierce).

### Edema factor adenylate cyclase assay.

A competition enzyme-linked immunoassay (Cell Signaling Technology, Danvers, MA) was used to determine the amount of cAMP generated in A431 cells upon exposure to EF. A431 cells (3.5 × 10^4^) were plated in a 96-well tissue culture plate and incubated with EF (50 nM) in the presence or absence of a PA variant (10 nM WT PA, PA^F427H^, mPA, or mPA-EGF). After 1 h, the medium was removed and cells were washed twice with 200 µl of ice-cold PBS. Adherent cells were lysed with 100 µl of 1× cell lysis buffer and incubated on ice for 10 min. Each cell lysis supernatant (50 µl) was combined with HRP-linked cAMP solution (50 µl), added to the cAMP assay plate, and incubated at room temperature for 3 h. Each well was then washed four times with 200 µl of 1× wash buffer, and 3,3′,5,5′-tetramethylbenzidine (TMB) substrate (100 µl) was added to each well for 10 min. Following the addition of STOP solution (100 µl), the absorbance of each well was read at 450 nm and used to estimate the amount of cAMP based on a standard curve. The amount of intracellular cAMP produced by EF in the presence and absence of each PA variant was plotted as a histogram where each bar represents the average of four experiments.

## SUPPLEMENTAL MATERIAL

Figure S1 Characterization of mPA-DTR. (A) Western blot analysis with anti-PA and anti-DTR antibodies demonstrating the presence of both PA and DTR in the purified mPA-DTR fusion. (B) A planar lipid bilayer was formed with 35 mM 1,2-diphytanoyl-*sn*-glycerol-3-phosphocholine (Avanti Polar Lipids, Alabaster, AL) in *n*-decane. mPA-DTR prepores (25 pM) were added to the *cis* compartment of the bilayer chamber (arrow 1). After the appropriate current increase, the *cis* compartment was perfused with approximately 10 ml of non-PA-containing buffer at a flow rate of ~3 ml/min to remove any free PA. Once the current was constant, LF_N_-DTA was added to the *cis* compartment (arrow 2), and its binding to PA channels was monitored by the decrease in conductance. The *cis* compartment was held at a constant voltage (Ψ) of 20 mV with respect to the *trans* compartment for the duration of the experiment. (C) CHO-K1 cells (3.5 × 10^4^) were exposed overnight to a range of concentrations of LF_N_-DTA in the presence of WT PA or mPA-DTR, with or without excess soluble DTR. Protein synthesis was determined by [^3^H]leucine incorporation. Each point on the curve corresponds to the average of three experiments. Download Figure S1, TIFF file, 0.3 MB
