# Supplementary figures and images for: Changing the Receptor Specificity of Anthrax Toxin
Source: mBio. 2012 May 1;3(3):e00088-12. doi: 10.1128/mBio.00088-12 (PMC3569862; doi:10.1128/mBio.00088-12)

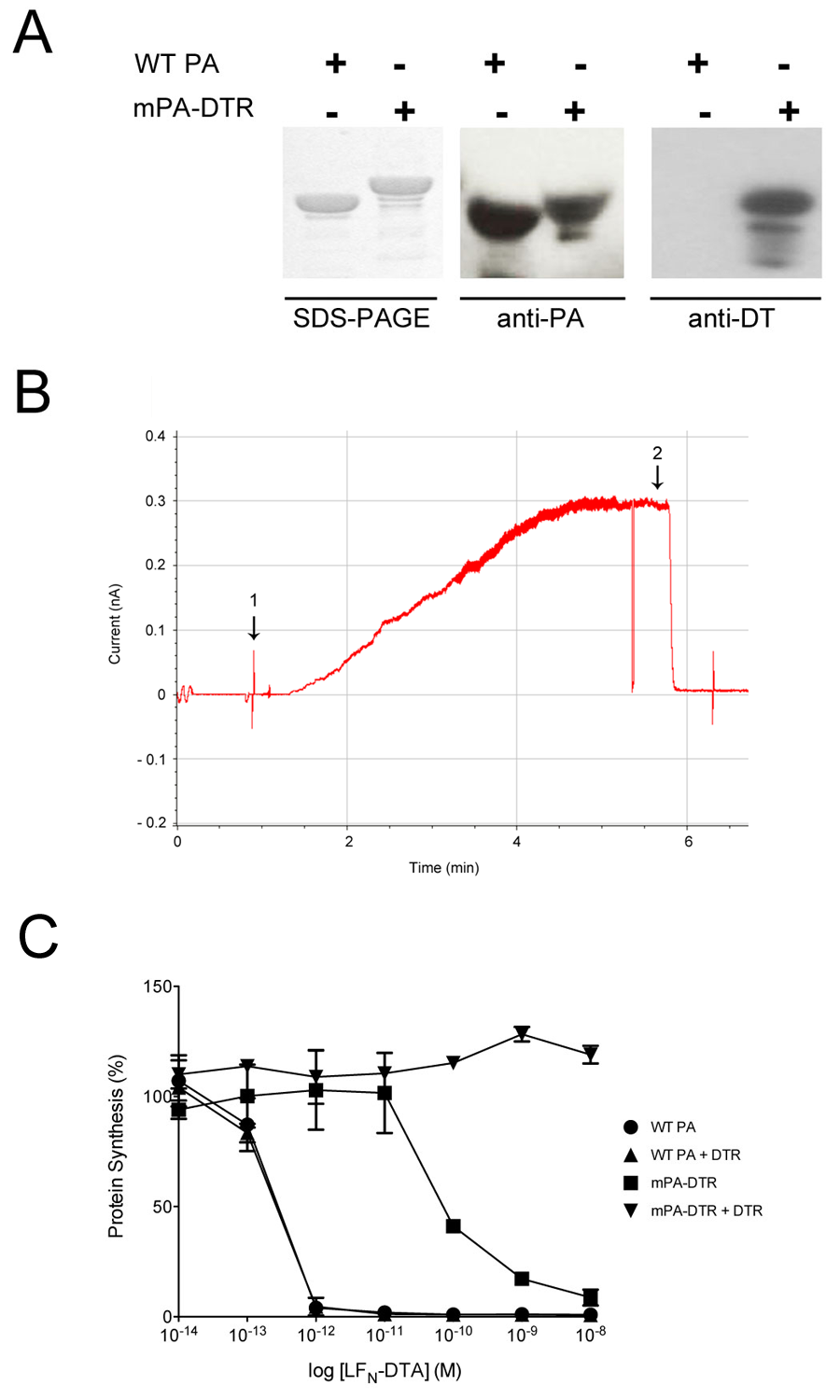

Supplement: Figure S1 — Characterization of mPA-DTR. (A) Western blot analysis with anti-PA and anti-DTR antibodies demonstrating the presence of both PA and DTR in the purified mPA-DTR fusion. (B) A planar lipid bilayer was formed with 35 mM 1,2-diphytanoyl-sn-glycerol-3-phosphocholine (Avanti Polar Lipids, Alabaster, AL) in n-decane. mPA-DTR prepores (25 pM) were added to the cis compartment of the bilayer chamber (arrow 1). After the appropriate current increase, the cis compartment was perfused with approximately 10 ml of non-PA-containing buffer at a flow rate of ~3 ml/min to remove any free PA. Once the current was constant, LFN-DTA was added to the cis compartment (arrow 2), and its binding to PA channels was monitored by the decrease in conductance. The cis compartment was held at a constant voltage (Ψ) of 20 mV with respect to the trans compartment for the duration of the experiment. (C) CHO-K1 cells (3.5 × 10^4^) were exposed overnight to a range of concentrations of LFN-DTA in the presence of WT PA or mPA-DTR, with or without excess soluble DTR. Protein synthesis was determined by [3H]leucine incorporation. Each point on the curve corresponds to the average of three experiments. Download [file mbo002121267sf01.tif]
